# New Putative Antimicrobial Candidates: *In silico* Design of Fish-Derived Antibacterial Peptide-Motifs

**DOI:** 10.3389/fbioe.2020.604041

**Published:** 2020-12-03

**Authors:** Hedmon Okella, John J. Georrge, Sylvester Ochwo, Christian Ndekezi, Kevin Tindo Koffi, Jacqueline Aber, Clement Olusoji Ajayi, Fatoumata Gnine Fofana, Hilda Ikiriza, Andrew G. Mtewa, Joseph Nkamwesiga, Christian Bernard Bakwo Bassogog, Charles Drago Kato, Patrick Engeu Ogwang

**Affiliations:** ^1^Pharm-Biotechnology and Traditional Medicine Center, Mbarara University of Science and Technology, Mbarara, Uganda; ^2^Department of Bioinformatics, Christ College, Rajkot, India; ^3^College of Veterinary Medicine, Animal Resources and Bio-Security, Makerere University, Kampala, Uganda; ^4^Biotechnology Engineering Department, V. V. P College of Engineering, Rajkot, India; ^5^Department of Bioinformatics, African Center of Excellence in Bioinformatics, University of Science, Technique and Technology, Bamako, Mali; ^6^Chemistry Section, Malawi Institute of Technology, Malawi University of Science and Technology, Thyolo, Malawi; ^7^International Livestock Research Institute, Kampala, Uganda; ^8^Center for Food and Nutrition Research, Institute of Medical Research and Medicinal Plants Studies, Yaounde, Cameroon

**Keywords:** antimicrobial, fish, peptides, putative, motifs

## Abstract

Antimicrobial resistance remains a great threat to global health. In response to the World Health Organizations’ global call for action, nature has been explored for novel and safe antimicrobial candidates. To date, fish have gained recognition as potential source of safe, broad spectrum and effective antimicrobial therapeutics. The use of computational methods to design antimicrobial candidates of industrial application has however, been lagging behind. To fill the gap and contribute to the current fish-derived antimicrobial peptide repertoire, this study used Support Vector Machines algorithm to fish out fish-antimicrobial peptide-motif candidates encrypted in 127 peptides submitted at the Antimicrobial Peptide Database (APD3), steered by their physico-chemical characteristics (i.e., positive net charge, hydrophobicity, stability, molecular weight and sequence length). The best two novel antimicrobial peptide-motifs (A15_B, A15_E) with the lowest instability index (−28.25, −22.49, respectively) and highest isoelectric point (p*I*) index (10.48 for each) were selected for further analysis. Their 3D structures were predicted using I-TASSER and PEP-FOLD servers while ProSA, PROCHECK, and ANOLEA were used to validate them. The models predicted by I-TASSER were found to be better than those predicted by PEP-FOLD upon validation. Two I-TASSER models with the lowest c-score of −0.10 and −0.30 for A15_B and A15_E peptide-motifs, respectively, were selected for docking against known bacterial-antimicrobial target-proteins retrieved from protein databank (PDB). Carbapenam-3-carboxylate synthase (PDB ID; 4oj8) yielded the lowest docking energy (−8.80 and −7.80 Kcal/mol) against motif A15_B and A15_E, respectively, using AutoDock VINA. Further, in addition to Carbapenam-3-carboxylate synthase, these peptides (A15_B and A15_E) were found to as well bind to membrane protein (PDB ID: 1by3) and Carbapenem synthetase (PDB: 1q15) when ClusPro and HPEPDOCK tools were used. The membrane protein yielded docking energy scores (DES): −290.094, −270.751; coefficient weight (CW): −763.6, 763.3 for A15_B and A15_E) whereas, Carbapenem synthetase (PDB: 1q15) had a DES of −236.802, −262.75 and a CW of −819.7, −829.7 for peptides A15_B and A15_E, respectively. Motif A15_B of amino acid positions 2–19 in Pleurocidin exhibited the strongest *in silico* antimicrobial potentials. This segment could be a good biological candidate of great application in pharmaceutical industries as an antimicrobial drug candidate.

## Introduction

Infections caused by drug resistant bacteria remain one of the leading causes of death worldwide (Martín-Rodríguez et al., 2016), as the potential of conventional antibiotics to combat such microbial infections fall (Tillotson and Zinner, 2017). Over 700,000 lives are lost to antimicrobial resistance annually and the number is projected to increase (O’Neill, 2014). The rate at which these microorganisms develop resistance has outpaced the rate of production of the current class of antibiotics in spite of the immense attempts by pharmaceutical industries for new antibiotics, thereby complicating the overall efforts (Huttner et al., 2013).

Several attempts like phage therapy (Moghadam et al., 2020), anti-biofilms agents (Pletzer and Hancock, 2016; Hamayeli et al., 2019), and the use of phytochemicals (Manuel et al., 2012) have been pipelined to prevent antimicrobial resistance. Antimicrobial peptides also known as host defensive proteins (HDPs) biologics are gradually gaining ground as far as countering multiple drug resistance is concerned (Fox, 2013). A case to note is Tyrothricin; the first peptide antibiotic to be clinically used in humans (Dubos, 1939). Since its discovery over six decades ago, no record of resistance has been reported against Tyrothricin (Atiye et al., 2014). Similarly, polymixin B and Colistin are among the only standing antibiotics for the treatment of multiple drug resistant bacteria including the notorious *Acinetobacter baumanni*, *Pseudomonas aeruginosa*, and *Klebsiella pneumoniae* as the last line antibiotics (Falagas and Kasiakou, 2005). Their ability to withstand resistance has been attributed to their non-specific mechanism of action, multiple target sites and presence of rare D-amino acids (Ageitos and Villa, 2016). They classically conform to the first mode of action by interfering with bacterial peptidoglycan cell wall biogenesis to ease cell membrane disruption (Sujeet et al., 2018; Hao et al., 2019) and as ligands for bacterial intracellular targets (Mahlapuu et al., 2016). Most antimicrobial peptides have generally recognized as safe (GRAS) status (Hancock and Scott, 2000), with little or no toxicity (Wang S. et al., 2016). These good attributes have led to an intensified search for novel peptide antibiotics from diverse forms of life.

Fish are capable of producing antimicrobial peptides of various classes including defensins, cathelicidins, hepcidins, histone-derived peptides, and piscidins (Masso-silva and Diamond, 2014; Kumar et al., 2018). These fish derived antimicrobial peptides are active against both fish and human pathogens (Hayek et al., 2013; Huan et al., 2020; Tiralongo et al., 2020). However, their low stability coupled with insufficient information about their structures has limited their pharmaceutical applicability (Okella et al., 2018), since information on protein structure and biological (motif) interaction are key for determining the stability of any active protein (Vaidya et al., 2018). Antimicrobial activity of peptides greatly relies on amino acid composition, structure and their physicochemical properties (Kêska and Stadnik, 2017). There are numerous experimentally validated fish-derived antimicrobial peptides. However, insights into the amino acid composition, peptide structure and the target interactions with motifs in these antimicrobial peptides are lacking and present a gap that needs to be understood. This gap can however be filled through the use of *in silico* approaches. In this study we report findings of motif design, target identification and target interactions with putative antimicrobial peptide motif derived from fish.

## Materials and Methods

### Study Design

This was an *in silico* study setup involving fishing out novel antimicrobial peptide motifs encrypted in 127 fish antimicrobial peptides on Antimicrobial Peptide Databases. Potential antimicrobial peptide motifs were then selected based on their physicochemical characteristics like hydrophobicity, stability, and molecular weight/size as well as sequence length. The best two antimicrobial peptide candidate-motifs were designed for their putative antimicrobial leads and docked against the known antimicrobial protein-targets to predict their potential mode of action.

### Retrieval of Antimicrobial Peptide Sequence

Out of the 127 existing antimicrobial peptide (AMP) sequences, a total of 24 naturally occurring peptides (<100 amino acid residues) of fish origin (Table 1), with well characterized antimicrobial activity were retrieved from Antimicrobial Peptide Database (APD3) using fish as the source organism at http://aps.unmc.edu/AP/tools.php (Retrieved on May 19th, 2019) (Wang G. et al., 2016).

**TABLE 1:** Retrieved fish-derived antimicrobial peptide.

**APD ID**	**Name of peptide**	**Source (spp.)**	**Amino acid length**	**AMP family**
AP00492	Misgurin	*Misgurnus anguillicaudatus*	21	Piscidin
AP00555	Parasin I	*Parasilurus asotus*	19	Not reported
AP00691	HFIAP-1	*Myxine glutinosa*	37	Cathelicidin
AP00692	HFIAP-3	*Myxine glutinosa*	30	Cathelicidin
AP01619	HbbetaP-1	*Ictalurus punctatus*	33	Not reported
AP01648	Pelteobagrin	*Pelteobagrus fulvidraco* R.	22	Not reported
AP01796	saBD	*Sparus aurata*	42	Defensin
AP02159	Chionodracine	*Chionodraco hamatus*	22	Piscidin-like
AP02521	PaLEAP-2	*Plecoglossus altivelis*	41	Not reported
AP02982	RP6	*Oplegnathus fasciatus*	15	Not reported
AP02983	RP7	*Oplegnathus fasciatus*	21	Not reported
AP00473	Piscidin 1	*Morone saxatilis*	22	Piscidin
AP00474	Piscidin 3	*Morone saxatilis*	22	Piscidin
AP02050	sb-Moronecidin	*Morone saxatilis*	23	Piscidin
AP00166	Pleurocidin	*Pleuronectes americanus*	25	Pleurocidin
AP02219	Cod-β defensin	*Gadus morhua*	38	Defensin
AP01713	CodCath	*Gadus morhua*	67	Cathelicidin
AP00537	SAMP H1	*Salmo salar*	30	Not reported
AP00411	Oncorhyncin II	*Oncorhynchus mykiss*	69	Not reported
AP00489	Hipposin	*Hippoglossus hippoglusus* L.	51	Not reported
AP00644	Pardaxin 4	*Pardachirus marmoratus*	33	Not reported
AP00302	Hepcidin	*Morone chrysops*	21	Hepcidin
AP02049	wb-Moronecidin	*Morone saxatilis*	*23*	Piscidin
AP02521	PaLEAP-2	*Plecoglossus altivelis*	*41*	Not reported

### Antimicrobial Peptide-Motif Design

To generate and identify potential antimicrobial peptide motifs, the retrieved sequences in *FASTA* file format were subjected to web-based Support Vector Machines (SVMs) algorithm based tool of Collection of Anti-Microbial Peptides (CAMP_*R*__3_) server (May, 2019)^1^ (Waghu et al., 2016). The generated motifs were then screened based on several physiochemical parameters (Torrent et al., 2012a). The choice of the physiochemical parameters took into account that of the already existing polycationic and amphipathic AMPs; Amino acid length (18 residues), positive net charge (+4 to +6), hydrophobicity (40 and 60%) and isoelectric point of up to 10 (Wang S. et al., 2016; Hincapié et al., 2018). Helical wheels for the generated motif sequences were determined using HeliQuest server^2^ at 18 amino acid window and one turn size (Gautier et al., 2008), so as to come up with cationic and hydrophobic amino acids, hydrophobicity and hydrophobic moment among other characteristics of the potential motifs (Torrent et al., 2012b). Furthermore, the instability of the putative peptides was checked using an *ExPASy* tool; *ProtParam*^3^, where an instability index above zero implies it’s an unstable peptide.

### Antimicrobial Peptide-Motif 3D Structure Prediction and Evaluation

Due to the shortness of the peptide sequences (<30 amino acids) coupled with the absence of their experimentally attained structure for templates, the three dimensional structure of putative peptide-motifs were predicted using the Iterative Threading Assembly Refinement (I-TASSER) server^4^ (Yang and Zhang, 2015). The peptides were modeled using protein templates identified by Local Meta-Threading Server (LOMETS) from the Protein Data Bank (PDB) library. LOMETS uses multiple threading approaches to align the query protein amino acid sequence against the PDB^5^. Template proteins with the highest sequence identity and lowest *Z*-score were used in the modeling exercise (Table 2). The best models were identified based on their c-scores. This score is calculated based on the significance of threading template alignments and the convergence parameters of the structure assembly simulations. It ranges from -5 to 2, where a lower score value indicates a highly confident model while the higher indicates the reverse. The peptide 3D structure prediction exercise was cross-validated using a web-based *de novo* peptide structure prediction tool, PEP-FOLD *v3.5*^6^ (Thévenet et al., 2012). Briefly the query peptide amino acid sequences in *FASTA* format were used as the input file sequences. The algorithm was set to run 100 simulations and the output models were ranked based on sOPEP energies of individual model, where the lower the energy the better the model. The best models for both peptides A15_A and A15_B from the two peptide structure prediction tools (I-TASSER and PEP-FOLD v3.5) were then analyzed for their quality. Validation of these peptides structure was carried out in three phases; First by using Protein Structure Analysis (ProSA) web-server^7^ (Wiederstein and Sippl, 2007) which predicts the query protein *z*-score, local model quality, and residue energy. The *Z*-score indicates the model quality by comparing the query protein *z*-score against the *z*-score of experimentally validated proteins available in the protein data bank (PDB). In the second phase, PROCHECK was then used to measure the stereo-chemical properties of the modeled peptide-motifs (Laskowski et al., 1993), and finally, Atomic Non-Local Environment Assessment (ANOLEA) web server^8^ was used to calculate the energy of the query protein and evaluate their heavy atomic Non-Local Environment (NLE) in each molecule (Melo et al., 1997).

**TABLE 2:** Template protein strictures used in the modeling exercise.

	**A15_B**					**A15_E**				
**SN**	**PDB-Id**	**Iden1**	**Iden2**	**Cov**	**N *Z*-score**	**PDB-Id**	**Iden1**	**Iden2**	**Cov**	**N *Z*-score**
1	2la2A	0.59	0.5	0.94	1.75	1rimA	0.28	0.28	1	1.66
2	6g65A	0.28	0.28	1	1.1	6mzcE	0.17	0.22	1	1.09
3	6cfz	0.45	0.28	0.61	1.02	1rimA	0.28	0.28	1	1.51
4	1tf3A	0.35	0.33	0.94	2.09	2la2	0.35	0.5	0.94	1.04
5	2kfqA	0.22	0.28	1	1.62	3bzlA	0.07	0.06	0.83	1.64
6	1rimA	0.24	0.22	0.94	1.07	2la2A	0.33	0.5	1	1.59
7	3jqhA	0.11	0.11	1	1.77	3t8sA	0.22	0.33	1	1.01
8	2jpkA	0.33	0.33	1	1.6	2pq4B	0.33	0.33	1	1.31
9	1p7aA	0.18	0.28	0.94	1.01	1be3K	0.22	0.28	1	1.56
10	1jlzA	0.39	0.39	1	1.74	2juiA	0.33	0.33	1	1.58

**Iden1 is the percentage sequence identity of the templates in the threading aligned region with the query sequence. Iden2 is the percentage sequence identity of the whole template chains with query sequence. Cov represents the coverage of the threading alignment and is equal to the number of aligned residues divided by the length of query protein. N Z-score is the normalized Z-score of the threading alignments. Alignment with a Normalized Z-score >1 mean a good alignment and vice versa.**

### Target Fishing

To identify the most probable target-proteins of the motifs, all the approved antibiotic targets in the *DrugBank* database (Law et al., 2014) at https://www.drugbank.ca/targets were fished using key words; target and antibiotics. The receptor proteins alongside their identities were later retrieved from Protein Data Bank (PDB) library.

### Molecular Docking Studies

The docking exercise was carried out on the top two potential AMP motifs against known protein drug targets. Docking was carried-out using the AutoDock VINA (Trott and Olson, 2019) on the DINC 2.0 Web server^9^ (Antunes et al., 2017). The docking was validated using two docking tools; Hierarchical flexible Peptide Docking (HPEPDOCK) and ClusPro (Kozakov et al., 2017; Zhou et al., 2018) for optimized protein-peptide interaction. HPEPDOCK predicts the protein-peptide interaction using the hierarchical algorithm between the protein and the peptide 3D structure while ClusPro performs a global docking procedure in four folds, motif-based prediction based on peptide conformation, rigid-body docking, scoring based on structural clustering; and final structure minimization. Briefly, the 3D structures of both the receptor protein (retrieved from PDB) and the modeled 3D peptide structures were the input files for both docking tools. Both ClusPro and HPEPDOCK docking were performed onto their respective web servers^10^^,^^11^.

## Results

### Sequence Retrieval

A total of 127 fish derived peptide sequences were retrieved out of which, 24 peptide sequences were qualified (Table 1). The average peptide-amino acid length was 32 residues (ranging from 15–69 residues). 20% of the retrieved peptide-sequences belonged to the cathelcidin family with 45.8% not reported. The target organisms of the retrieved peptides ranged from bacteria to yeast and fungi.

### Antimicrobial Peptide Motif Design

A total of 361 peptide-motif sequences were designed from the qualified sequences which had suitable physico-chemical properties *viz.* mean hydrophobicity (*H*_*m*_) greater than 0.3 (based on Fauchere and Pliska scale) (Fauchere and Pliska, 1983), net charge of + 4 and above, low instability index below zero, high antimicrobial probability were qualified. Seven peptide-motifs (Table 3), from which two peptide-motifs (A15_B and A15_E) with the highest stability (least instability index −28.25, −22.49, respectively) and highest antimicrobial probability (0.982) were selected for docking studies. Both peptides were found to be from the sequence of Pleurocidin; an AMP secreted by a winter flounder fish, *P. americanus* located between amino acids 2–19 and 5–22, respectively.

**TABLE 3:** Physicochemical properties of peptides sourced through *in silico* analysis.

**Peptide**	**Sequence**	**Charge**	**H (%)**	**H_*m*_**	**μ H_*r*_**	**pI**	**Ii**	**MW (Da)**	**A_*p*_**
A15_B	WGSFFKKAAHVGKHVGKA	+4	44.44	0.303	0.508	10.48	–28.25	1955.30	0.982
A15_E	FFKKAAHVGKHVGKAALT	+4	50.00	0.307	0.347	10.48	–22.49	1910.30	0.982
A20_Q	RSSRAGLQFPVGRVHRLL	+4	44.44	0.342	0.349	12.48	85.86	2049.41	0.741
A20_R	SSRAGLQFPVGRVHRLLR	+4	44.44	0.342	0.349	12.48	72.15	2049.41	0.729
A20_U	AGLQFPVGRVHRLLRKGN	+ 4	44.44	0.314	0.380	12.30	41.32	2018.40	0.917
A20_V	GLQFPVGRVHRLLRKGNY	+4	44.44	0.350	0.344	11.72	41.32	2110.50	0.816
A20_W	LQFPVGRVHRLLRKGNYA	+4	50.00	0.367	0.346	11.72	54.47	2124.52	0.740

**H, Hydrophobicity; Hm, mean hydrophobicity; μHr, relative hydrophobic moment; Ii, instability index; MW, molecular weight; A_*p*_, Antimicrobial probability.**

### Peptide Motifs 3D Structure Prediction and Evaluation

The I-TASSER modeling returned five models for each modeled peptide motif (A15_B and A15_E),while the PEP-FOLD prediction returned 10 models. The best I-TASSER models had a negative c-score. I-TASSER Model-1 for both peptides (A15_B and A15_E) had the best c-score of -0.10 and -0.03, respectively (Table 4). On the other hand PEP-FOLD model1 for both peptides (A15_B and A15_E) were recognized as the best model with the lowest sOPEP energy of −25.1325 and −25.4534 and Apollo predicted melting temperature (tm) score of 0.703 and 0.714, respectively. The Model1_A15_B and Model1_A15_E for both I-TASSER and PEP-FOLD were characterized as the best models from both tools, thus selected for model structure analysis.

**TABLE 4:** Top 5 output peptide structure prediction models from i-TASSER, PEP-FOLD, and their model evaluation.

**Models**	**iTASSER output modelC-score**		**PEP-FOLD output model scores**			
	**A15_B**	**A15_E**	**A15_E**		**A15_B**	
			**sOPEP**	**tm**	**sOPEP**	**tm**
Model1	−0.10	−0.03	–25.4534	0.703	–25.1325	0.714
Model2	−5	−5	–25.3043	0.661	–25.0347	0.740
Model3	−5	−5	–25.2665	0.716	–25.0096	0.764
Model4	−5	−5	–25.0894	0.694	–24.8657	0.760
Model5	−5	−1.76	–24.895	0.670	–24.7082	0.739

**FIGURE 1:**
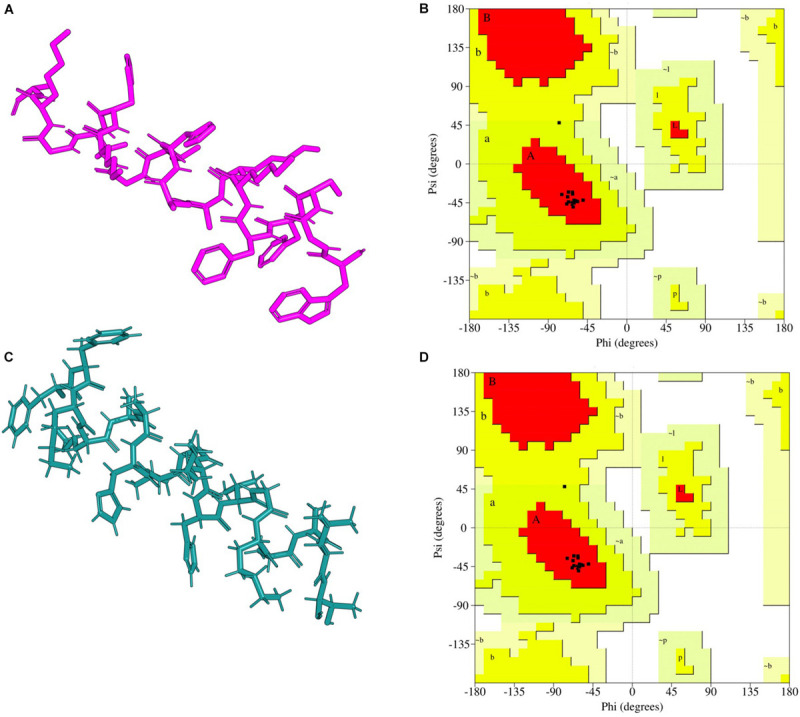
I-TASSER predicted peptide 3D structure homology models and their Ramachandran validation plots. **(A)** A15_B peptide-motif, **(B)** Ramachandran plot for A15_B peptide-motif, **(C)** A15_E peptide-motif, **(D)** Ramachandran plot for A15_E peptide-motif. Peptide-motif A15_B had 12 amino acids sequences in the allowed region while peptide-motif A15_E had 13 amino acid in the favorable region. Both peptide-motifs had no amino acid sequence in the disallowed region. The cartons were rendered in *Edu PyMOL*.

**FIGURE 2:**
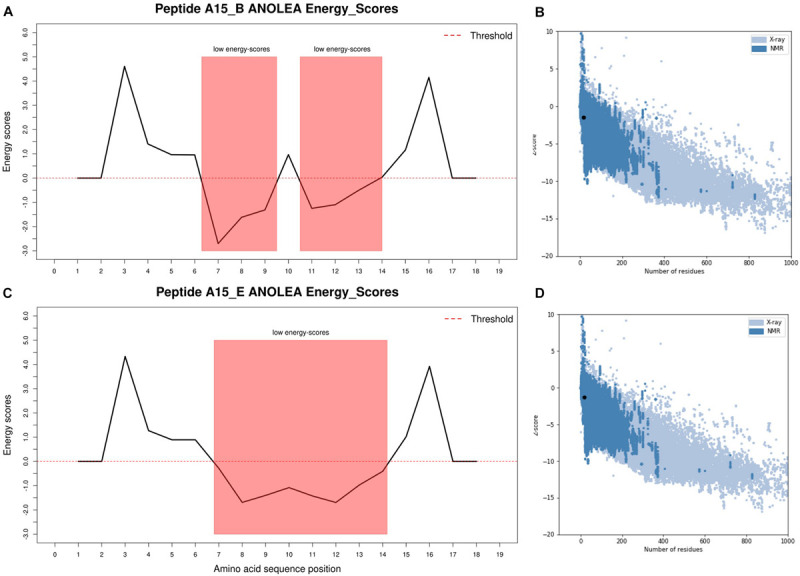
I-TASSER predicted peptide 3D structure ANOLEA and ProSA validation plots. **(A)** Peptide A15_B ANOLEA energy score, **(B)** Peptide A15_B ProSA z-score, **(C)** Peptide A15_E ANOLEA energy score, **(D)** Peptide A15_B ProSA z-score. ANOLEA validation showed that 33.3 and 44.5% of peptide A15_B and A15_E had their amino acid residues in the favorable regions (low energy scores highlighted in red). Peptide motifs A15_B and A15_E had *z*-scores of −1.5 and −1.27, respectively, and were within the normal *z-*score of experimentally validated proteins. The ANOLEA plots were generated in R using latticeExtra package.

The Ramachandran plot analysis indicates that I-TASSER Model1_A15_E had 13 residues in the most favorable region and 1 in the additional allowed region. None of the Model1_A15_E peptide-motif residues were in the disallowed region. Similarly, I-TASSER Model1_A15_B had 12 residues in the most favorable region, 1 in the additional allowed region with none in disallowed region (Figure 1; Laskowski et al., 1993). On the other hand, PEP-FOLD model1_A15_B had 13 residues in the favorable region while PEP-FOLD model1 A15_E had 14 residues in the favorable region. In addition, a cross-validation with ProSA, showed that I-TASSER models had a *z*-score of -1.5, -1.27 against A15_B and A15_E, while a *z*-score of -1.44, -1.5 were observed for PEP-FOLD A_15_B and A_15_E models, respectively. All model *z*-scores were in the same range with the z-score of experimentally validated proteins, thus considered to be accurate. Likewise, ANOLEA showed that majority of I-TASSER models (33.3 and 44.5% for model A15_B and A15_E, respectively) had amino acid residues of the peptide chain in a favorable energy environment (with low energy-scores) (Figure 2) while PEP-FOLD model A15_B and A15_E had 22.2 and 55.7% of amino acid residues with low energy. I-TASSER model1 for both A15_B and A15_E show to be the best peptide structures and they were selected for docking exercise.

### Target Fishing

A total of 28 targets were fished from the *DrugBank* database, out of which 18 had experimentally determined structures deposited at PDB (Table 5). Majority of the structures (83.3%) were determined using X-ray diffraction with only one structure (C-1027) determined using solution Nuclear Magnetic Resonance (NMR).

**TABLE 5:** Antimicrobial target proteins used in the docking exercise.

**Protein name**	**PDB Id**	**Classification**	**Organism**	**Method**
C-1027	1hzl	Antibiotic	*Streptomyces globisporus*	Solution NMR
Tyrosine aminomutase	3kdy	Lyase	*Streptomyces globisporus*	X-ray diffraction
50s ribosomal protein l32	6qul	Antibiotic	*Escherichia coli*	Electron microscopy
Carbapenam synthetase	1q15	Biosynthetic protein	*Pectobacterium carotovorum*	X-ray diffraction
Iron(3 +)-hydroxamate-binding protein fhud	1esz	Metal transport	*Escherichia coli*	X-ray diffraction
Fhua	1by3	Membrane protein	*Escherichia coli*	X-ray diffraction
Neocarzinostatin	1nco	Antibacterial and antitumor protein	*Streptomyces carzinostaticus*	X-ray diffraction
Protein phzg	1ty9	Oxidoreductase	*Pseudomonas fluorescens*	X-ray diffraction
Lipocalins	1nyc	Hydrolase inhibitor	*Escherichia coli*	X-ray diffraction
D-alanyl-d-alanine carboxypeptidase	6osu	Hydrolase	*Francisella tularensis* subsp. *Tularensis schu* s4	X-ray diffraction
Beta-hexosaminidase	4g6c	Hydrolase	*Burkholderia cenocepacia* j2315	X-ray diffraction
Mexa of the multidrug transporter	1vf7	Membrane protein	*Pseudomonas aeruginosa*	X-ray diffraction
S/t protein kinase pkng	4y0x	Transferase	*Mycobacterium tuberculosis* h37rv	X-ray diffraction
Bacterial 45srbga ribosomal particle class a	6pvk	Ribosome	*Bacillus subtilis*	Electron microscopy
Neocarzinostatin	1nco	Antibacterial and antitumor protein	*Streptomyces carzinostaticus*	X-ray diffraction
Carbapenam	4oj8	Oxidoreductase	*Pectobacterium carotovorum* subsp. Carotovorum	X-ray diffraction
Vancosaminyl transferase	1rrv	Transferase/antibiotic	*Amycolatopsis orientalis*	X-ray diffraction

### Molecular Docking

Docking exercise with AutoDock VINA revealed that both peptide-motifs (A15_B and A15_E) were able to bind with low docking energies (ranging from -8.80 to -5.80 Kcal/mol) indicating their fairly high affinity with the selected antimicrobial target protein (Table 6). The best docking energy, however, was observed against *vancosaminyl transferase* protein (PDB ID; 1rrv, docking energy (DE); −8.20, −7.60 Kcal/mol), *Beta-hexosaminidase* protein (PDB ID; 4g46 DE; −7.90, −7.70 Kcal/mol), membrane protein (PDB: 1by3 DE; −7.3, −7.3), and *carbapenam* protein (PDB ID; 4oj8 DE; −8.80, −7.80 Kcal/mol) against peptide-motif A15_B and A15_E, respectively (Table 6). The affinity of peptide-motifs A15_B and A15_E was highest within chains of the target proteins (PDB ID 1rrv, 4g6c, and 4oj8). Docking validation with HPEPDOCK shows that membrane protein (PDB: 1by3) and Carbapenem synthetase had the highest docking potential to peptide A15_B and A15_E with a docking energy score of −290.094, −270.751 against protein 1by3 and −236.802, −262.75 against 1q15, respectively. Likewise, docking with ClusPro further indicated that membrane protein and carbapenem synthetase had the highest chance to bind to peptide A15_B and A15_E with a coefficient weight of −763.6, −763.3 against protein 1by3 and −819.7, −829.7 against peptide A15_B and A15_E, respectively. Carbapenam synthetase (PDB ID; 4oj8) which had the lowest docking energy against the two peptides was found to be among the targets with lowest docking energies scores of (-221.657 and −196.952) against peptide A15_B and A15_E using HPEPDOCK. However, this protein had the lowest coefficient weight score of −681.2 and −66.8 against peptide A15_B and A15_E using ClusPro, respectively. Peptide motif A15_B which had the lowest instability index (highest stability) also showed a relatively higher binding affinity than its counterpart A15_E (Table 6) in all the 3 docking methods, except with protein 1by3 where peptide A15_E had a lower docking energy score than A15_B using HPEPDOCK.

**TABLE 6:** Docking energies and score of ligand A15_B, A15_E against the Antimicrobial target proteins using Autodock Vina, HPEPDOCK, ClusPro.

**DB ID**	**Center AA**	**Energies with AutoDock VINA (Kcal/mol)**		**Energy scores with HPEPDOCK**		**Coefficient weight score with ClusPro**	
		**A15_E**	**A15_B**	**A15_E**	**A15_B**	**A15_E**	**A15_B**
1by3	HIS-89	−7.30*	−7.30*	−290.094*	−270.751*	−763.6*	−763.3*
1e5z	PHE-274	–5.80	–6.40	–201.893	–202.313	–652.9	–766.9
1hzl	GLN-35	–5.40	–5.40	–192.021	–181.348	–594.3	–617.6
1kny	GLN-168	–7.10	–7.20	–186.724	–198.732	–767.7	–802.2
1nco	ALA-2	–6.20	–6.70	–199.495	–183.348	–678.9	–753.1
1nyc	TRP-31	–6.70	–6.60	–216.461	–206.614	–651.8	–791.5
1q15	ARG-50	–7.00	–6.80	−236.802*	−262.750*	−819.7*	−829.7*
1rrv	ALA-265	−7.60*	−8.20*	–208.564	–179.493	–761.8	–769.3
1ty9	VAL-108	–6.90	–6.60	–221.560	–196.827	–652.9	–677
3kdy	ASP-366	–6.10	–6.10	–233.213	–208.320	–663.6	–721.9
4g6c	HIS-158	−7.70*	−7.90*	–182.505	–213.155	–602.0	–700.4
4oj8	ALA-144	−7.80*	−8.80*	−221.657*	−196.952*	–681.2	–666.8
6osu	VAL-32	–6.20	–6.10	–182.232	–198.953	–511.7	–610.1

AA, Amino acid. *Proteins with the lowest docking energies. Lowest energies against protein with the highest probability to dock to peptide A15_B and A15_E.

## Discussion

The present study demonstrates that an online Support Vector Machines (SVMs) algorithm effectively localizes motifs of potentially best antimicrobial activity within a peptide. This technique is vital in enhancing the antimicrobial activity of peptides especially on resistant strains including *Pseudomonas aeruginosa* (Torrent et al., 2012c). The strength of this study is hinged on its ability to generate very many peptide fragments and being able to systematically sieve them based on their physicochemical parameters to arrive at the best candidates. However, the number of peptide templates used was small 24 (0.77%) compared to a total of 3,105 antimicrobial peptides in the antimicrobial peptides database (accessed on 01.08.2019). This is due to the fact that this study focuses only on “experimentally validated” peptides even so, only 127 fish antimicrobial peptides are present at the database.

Out of the 361 peptide motifs generated, the most active with the highest *in silico* antimicrobial probability of 0.982 (A15_B and A15_E) were both from Pleurocidin; an AMP secreted by flatfish, *Pleuronectes americanus* that largely inhabits soft muddy to moderately hard bottoms of marine waters. Even so, motif A15_B proved to be much more stable (instability index −28.25), rendering it the best fragment designed. When docked with AutoDock VINA, A15_B continued as the best designed peptide motif yielding the highest binding energy (−8.80 Kcal/mol) and highest number of hydrogen bond interactions (3) on Carbapenam-3-carboxylate synthase target. This indicates the motif (A15_B) binds spontaneously onto Carbapenam-3-carboxylate synthase target without consuming energy (Meng et al., 2011). Moreover, docking with HPEPDOCK and ClusPro further indicated that Carbapenam synthetase protein (PDB: 1Q15) alongside a Membrane proteins (PDB: 1by3) and Carbapenam-3-caboxylate protein (PDB: 4oj8) are among the proteins with highest binding potentials to peptide motif A15_B. However, Carbapenam-3-caboxylate protein yielded the least Docking energy when compared to the Membrane proteins and carbapenam synthetase and Carbapenam synthetase protein. Carbapenam-3-carboxylate synthase is responsible for the biosynthesis of the naturally occurring β-lactam antibiotics in bacteria (Stapon et al., 2003). The enzyme catalyzes the ATP-dependent formation of (3S,5S)-carbapenam-3-carboxylate from (2S,5S)-5-carboxymethylproline in *Pectobacterium carotovorum* (Gerratana et al., 2003). Therefore, the binding of the designed peptide motif A15_B is likely to activate Carbapenam-3-carboxylate synthase to synthesis amass of natural antibiotic that destroys the bacteria (Samantha et al., 2007), a phenomenon that can be explored for novel therapeutics. However, being a novel motif on amino acids of positions 2–19 of Pleurocidin, this study could hardly access preceding studies to match the complex binding affinity.

An important but unanswered question is how these peptides can be optimized for a good platform particularly in drug discovery where the nature and properties of potential hits can be understood specifically on how best they can be modified into useful leads as antimicrobials in the fight against drug resistance. Ultimately, efforts are underway for better ways to handle such small fragments on benches to ascertain the *in vitro* and *in vivo* efficacy in low resource facilities.

## Conclusion

This study revealed that the motifs (A15_B) of amino acid positions 2-19 in Pleurocidin secreted by a winter flounder fish, *Pleuronectes americanus* as the best antimicrobial potentials. This segment is among the promising biological candidates that could be of great application in pharmaceutical and nutraceutical industries as virtual tools show great potentials in drug development even in the absence of large investment laboratory equipment. However, further studies focused on synthesized peptides would be helpful.

## Data Availability Statement

The original contributions presented in the study are included in the article/supplementary material, further inquiries can be directed to the corresponding author/s.

## Author Contributions

HO, SO, and CN designed and implemented the study. JA, CA, HI, FF, JN, CK, CB, PO, HO, AM, JG, and KK performed the experiments and data analysis. All authors participated in writing and proofreading the manuscript and approved the final manuscript for publication.

## Conflict of Interest

The authors declare that the research was conducted in the absence of any commercial or financial relationships that could be construed as a potential conflict of interest.

## Footnotes

1 http://www.camp.bicnirrh.res.in

2 https://heliquest.ipmc.cnrs.fr/cgi-bin/ComputParams.py

3 https://web.expasy.org/protparam/

4 https://zhanglab.ccmb.med.umich.edu/I-TASSER/

5 http://www.rcsb.org/

6 https://bioserv.rpbs.univ-paris-diderot.fr/services/PEP-FOLD3/

7 https://prosa.services.came.sbg.ac.at/prosa.php

8 http://melolab.org/anolea/

9 http://dinc.kavrakilab.org/

10 https://bioserv.rpbs.univ-paris-diderot.fr/services/pepATTRACT/#docking-performance

11 https://cluspro.bu.edu/
